# The Prevalence of low back pain in Africa: a systematic review

**DOI:** 10.1186/1471-2474-8-105

**Published:** 2007-11-01

**Authors:** Quinette A Louw, Linzette D Morris, Karen Grimmer-Somers

**Affiliations:** 1 Division of Physiotherapy, Faculty of Health Sciences, Stellenbosch University, PO Box 19063, Tygerberg, 7505, South Africa; 2 Division of Health Sciences, University of South Australia, City East Campus, North Tce, Adelaide 5000, Australia

## Abstract

**Background:**

Low back pain (LBP) is the most prevalent musculoskeletal condition and one the most common causes of disability in the developed nations. Anecdotally, there is a general assumption that LBP prevalence in Africa is comparatively lower than in developed countries. The aim of this review was to systematically appraise the published prevalence studies conducted on the African continent to establish the prevalence of LBP in Africa.

**Methods:**

A comprehensive search was conducted in April 2006. The following databases PEDro, Psychinfo, Science Direct, SportsDiscus, PubMed, CINAHL, Biblioline Pro-African Wide NiPAD and SA ePublications were individually searched using specifically developed search strategies for epidemiological research conducted on LBP amongst the African population. Two reviewers independently evaluated the methodological quality of the studies reviewed.

**Results:**

A total of 27 eligible epidemiological studies were included in this review. The majority of the studies (63%) were conducted in South Africa (37%) and Nigeria (26%). The most common population group involved workers (48%), while scholars comprised 15% of the population. 67% of the studies were found to be methodologically sound, and the LBP prevalence of these were analyzed. The mean LBP point prevalence among the adolescents was 12% and among adults was 32%. The average one year prevalence of LBP among adolescents was 33% and among adults was 50%. The average lifetime prevalence of LBP among the adolescents was 36% and among adults was 62%.

**Conclusion:**

The findings support the global burden of disease of LBP, in addition to suggesting that LBP prevalence among Africans is rising and is of concern. Further research into the most effective strategies to prevent and manage LBP in Africa is warranted.

## Background

The health of Africans is of global concern, as improvement in health outcomes observed in most Western countries over the past few decades has not been achieved in Africa [1]. This has been attributed more recently to the negative impact of the HIV and AIDS epidemic, reflecting both the focus shift of health interventions, and funding directions in health research [1]. Africa accounts for about 14% of the world's population, and it is also the poorest continent, bearing about 40% of the global burden of disease [1,2]. A positive causal relationship between income and health is well recognized internationally, in which a higher income promotes good health by the economical ability to access clean water and sanitation, good nutrition and good quality health services [2]. Lack of access to these resources consequently predisposes communities to a greater prevalence of disease and disability [3]. Socioeconomic constraints in Africa therefore underpin the higher prevalence of many diseases and disabilities [1,2].

The global prevalence of general disability is highest in sub-Saharan Africa [4]. The etiology of disability is multi-factorial and varies between different parts of the world [4]. The most apparent difference in disability prevalence is between the developed and developing worlds [5], with the most frequent cause of disability being musculoskeletal disorders [6]. The difference in disability prevalence between the developed and developing worlds is one example of global differences in health. Musculoskeletal disorders accounts for about 4.3% of disability life adjusted years (years living with disability) in the developed world, whilst it is reported as accounting for approximately 1% in the developing world [1]. Pain and loss of function associated with musculoskeletal conditions primarily leads to disability [7]. The four major musculoskeletal conditions leading to disability include osteoarthritis, rheumatoid arthritis, osteoporosis and low back pain (LBP) [7].

LBP is the most prevalent musculoskeletal condition and the most common cause of disability in developed nations [7]. The lifetime prevalence of LBP (at least one episode of LBP in a lifetime) in developed countries is reported to be up to 85% [8]. LBP results in significant levels of disability, producing significant restrictions on usual activity and participation, such as an inability to work [9]. Furthermore, the economic, societal and public health effects of LBP appear to be increasing. LBP incurs billions of dollars in medical expenditures each year [10] and this economic burden is of particular concern in poorer nations such as Africa, where the already restricted health care funds are directed toward epidemics such as HIV and AIDS [8].

A review of research publications on LBP suggests that most research has been conducted in the developed world, where little racial heterogeneity exists [11]. Racial, economic and social homogeneity is not a feature of Africa, a developing country. It is logical, therefore, to argue that genetic diversity, and differences in social structure and economics between the developed and developing nations, may underlie reported differences in the prevalence of LBP [6]. Other African-specific factors such as the HIV and AIDS epidemic, types of work tasks and poor nutrition may also influence LBP prevalence among Africans [1].

The literature on the epidemiology of LBP is accumulating, but for the most part, studies are restricted to high-income countries, therefore little is known about the epidemiology of LBP in the rest of the world [12]. In developed countries such as the United States of America (USA) and Australia, LBP prevalence ranges from 26.4% to 79.2% [13,14]. There appears to be a general (albeit anecdotal) assumption that LBP prevalence in Africa is lower than that reported in the developed nations [15-17]. A systematic review into the global prevalence of LBP by Walker in 2000, identified that of the 56 included studies, only 8% were conducted in developing countries, with only one study conducted in Africa [8]. The lack of information on the prevalence of LBP in developing countries is therefore a significant shortcoming [8,17], particularly as it is predicted that the greatest increases in LBP prevalence in the next decade will be in developing nations [6]. Understanding prevalence and causality of LBP in developing nations such as Africa may assist understanding of global LBP causes and management [8,17], and will determine whether the factors differ in socio-cultural characteristics [17].

To our knowledge, no systematic review reporting on the prevalence of LBP on the African continent exists. The aim of this review was therefore to systematically appraise peer-reviewed published disease prevalence studies conducted on the African continent, in order to ascertain whether LBP is of concern among Africans, as it is globally. This review considered the methodological quality of the relevant literature in order to identify opportunities for improvement in research practices, as well as to establish a way forward for high quality research in this area in Africa.

## Methods

This review was part of a larger, more comprehensive study which investigated the prevalence of cervical, thoracic and lumbar spine pain (or LBP) in Africa. The LBP epidemiological findings are reported in this paper.

The specific objectives of this element of the review were:

1. To determine the prevalence of LBP on the African continent.

2. To describe the primary risk factors of LBP among Africans living on the African continent.

3. To critically appraise the methodological quality of the prevalence studies with a view to identifying opportunities to improve future research quality.

The following terms and definitions were applied to this review:

• ***Adolescent***: individual aged 11–19 years old.

• ***Adult***: individual aged 20 years and older.

• ***Low back pain***: Pain experienced in the lumbar region of the spine.

• ***Musculoskeletal condition***: Affecting the muscles and/or skeleton of the spinal column.

• ***Africa: ***All countries located on the African continent.

• ***Prevalence: ***the total number of cases of a disease in a given population at a specific time.

### Search Strategy

A comprehensive search was conducted in April 2006 in all accessible library databases of published research reports available at the Stellenbosch University Medical Library. No date limit was applied to any of the databases searched, and thus each database was searched since its inception. The electronic databases included: PEDro (1929 to present), Psychinfo (1806 to present), Science Direct (1823 to present), SportsDiscus (1800 to present), PubMed (1950 to present), CINAHL (1982 to present), Biblioline Pro-African Wide NiPAD (19^th ^century to present) and SA ePublications (19^th ^century to present).

Each database has its own indexing terms and functions, and therefore different search strategies were developed for each database by two of the authors. The main search terms were back pain, low back pain, spine, physiotherapy, Africa, epidemiology, prevalence and low back pain rehabilitation. In PubMed, medical subject headings (MeSH) terms were used where possible, with Boolean operators. The search strategies for remaining databases included synonyms of the main search terms. The search strategies are illustrated in Appendix A. Manual searching of journals not indexed in electronic databases was considered, however, all issues of the African journals were not available. This method was thus discarded as it would be difficult to replicate. Secondary searching (or PEARLing) was however undertaken, whereby the reference lists of the selected articles were reviewed for additional references not identified in the primary search.

The titles and abstracts of the all identified literature were screened by two reviewers independently (LM and QL) using the inclusion criteria below. The full text of all potentially relevant articles were retrieved and screened by the same two reviewers using the same criteria, in order to determine the eligibility of the paper for inclusion in the review.

### Inclusion criteria

The inclusion criteria were that the studies reported on epidemiological research, and were conducted on the African continent. The prevalence of LBP should be the study focus, in subjects of adolescent and adult age groups, any race and any gender. Studies could be written in either the English or French language, as these are the primary languages used in African academic publications.

### Methodological appraisal

The methodological quality critical appraisal tool used in a systematic review into the prevalence of global LBP (reviewing literature from 1966 to 1998) was applied to this review [8] (See Table 1). This tool uses three methodological tests containing 12 criteria for prevalence studies, which examine representation of the target population, data quality and definition of the LBP problem. All studies were independently appraised by two of the authors (LM and QL). Differences in opinion between the reviewers were discussed until consensus was reached.

**Table 1:** The critical appraisal tool [8]

	**A: Is the final sample representative of the target population?** 1. At least one of the following must apply in the study: an entire target population, randomly selected sample, or sample stated to represent the target population. 2. At least one of the following: reasons for nonresponse described, nonresponders described, comparison of responders and nonresponders, or comparison of sample and target population. 3. Response rate and, if applicable, drop-out rate reported.
	**B: Quality of the data?** 4. Were the data primary data of low back pain or was it taken from a survey not specifically designed for that purpose? 5. Were the data collected from each adult directly or were they collected from a proxy? 6. Was the same mode of data collection used for all subjects? 7. At least one of the following in case of questionnaire: a validated questionnaire or at least tested for reproducibility. 8. At least one of the following in the case of an interview: Interview validated, tested for reproducibility, or adequately described and standardized. 9. At least one of the following in the case of an examination: Examination validated, tested for reproducibility, or adequately described and standardized.
	**C: Definition of low back pain (LBP)** 10. Was there a precise anatomic delineation of the lumbar area or reference to an easily obtainable article that contains such specification? 11. Was there further useful specification of the definition of LBP, or question(s) put to study subjects quoted such as the frequency, duration or intensity, and character of the pain. Or was there reference to an easily obtainable article that contains such specification? 12. Were recall periods clearly stated: e.g., 1 week, 1 month or lifetime?

### Evidence hierarchy

The hierarchical system of evidence as described by Sackett et al (2000) was used to determine the level of evidence of the eligible and included studies [18] (Table 2). The level of evidence is a reflection of the degree to which bias has been considered within the study design [18]. Prevalence studies are epidemiological studies, and thus the studies sought in this systematic review should be found at Level 3 evidence in this hierarchy of evidence.

**Table 2:** Hierarchy of evidence [18]

Level 1	Meta-analysis of randomized controlled clinical trials
Level 2a	One randomized controlled clinical trial (RCT)
Level 2b	One non-randomized, or non-controlled, or non-blinded clinical trial
Level 3	Observational studies
Level 4	Pre-post test clinical trials
Level 5	Descriptive studies
Level 6	Anecdotal evidence

### Data extraction

Data was extracted into purpose-built MS Excel sheets from each relevant included study on author, year of publication, African country, study design, sample size, age, gender, study setting, data collection period, definition of LBP, LBP recall time period, severity classification and rate, reliability and validity of measurement tools, statistical tests, LBP point prevalence, LBP one year prevalence, LBP lifetime prevalence, risk factors and odds ratios for risk factors, LBP management and clinical implications or study recommendations.

### Data analysis

In order to compare the prevalence statistics reported in the included studies, the primary elements for homogeneity of data were analyzed. The essential quality reporting elements were established by the authors, which included information on gender, age and recall period [8]. Acceptable methodological quality was determined as the central tendency of the frequency distribution of methodological scores. The LBP prevalence data extracted from the methodologically sound studies were analyzed. Sensitivity analysis of the studies not found to be methodologically sound was done to determine if there would have been any difference in the results, had these studies been included for analysis.

## Results

**Figure 1:**
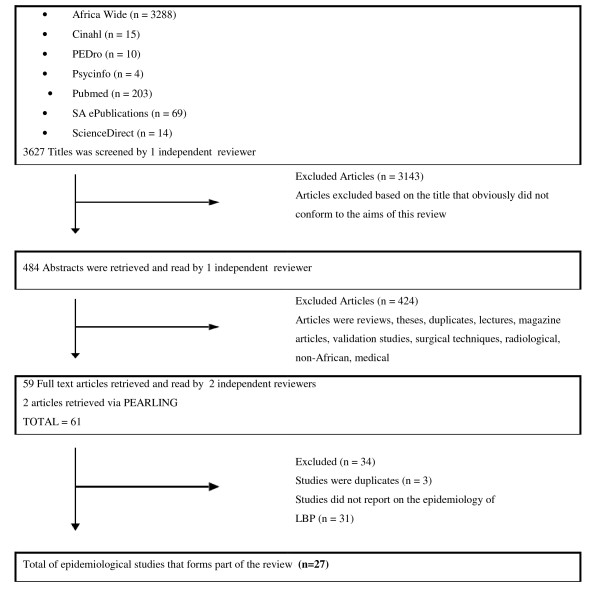
Database search results.

The comprehensive search for published epidemiological research into cervical, thoracic and lumbar spine pain (or LBP) conducted on the African continent yielded 3627 hits, of which 3143 articles were excluded as the title and/or the country of publication did not conform to this review's objectives. As the current review focused solely on the prevalence of LBP in Africa, studies which reported on cervical and thoracic spine pain were not included. Two additional relevant studies were included via the PEARLing method. Consequently, 27 eligible studies were included in this review [11,15,16,19-42]. The database search method and results are depicted in figure 1.

### Evidence hierarchy

As anticipated, all studies identified as eligible for inclusion in this review were epidemiological studies, denoted as Level 3 evidence in the hierarchy of evidence outlined in Table 2.

### General description of the studies reviewed

Descriptive data extracted from the 27 included studies is reported as an overview summary (Table 3). Twenty-two of the 27 (83%) studies were recent (published in and after the year 2000). The majority of the studies (63%) were from two African countries (South Africa (37%) and Nigeria (26%)). The remaining studies (37%) were conducted in Algiers, Zaire, Togo, Senegal, Mozambique, Tunisia, Uganda and Nairobi. Questionnaires were the common data collection tools in most studies. An examination was used in only one study [19]. The most common population on which research had been conducted was 'workers' (48%), while 'scholars' comprised 15% of the investigated population. 'Sporting participants' were only included in one study, conducted in South Africa on cricket players [20]. The sample sizes varied from 69 to 9065. The response rate varied from 53% to 100% in the included studies that reported it. Four studies did not report on any response rates [19,21-23]. The mean response rate for those studies which did report on the response rate was 88% (SD 16,08%). Seven of the studies investigated a rural population [11,21,22,24-26,41], while two studies [27,28] reported on a combined urban and rural population. The majority of the studies (67%) reported on the LBP prevalence in urban populations. The recall periods for LBP varied from point-, one year- and lifetime prevalence.

**Table 3:** Summary of reviewed studies (Q = questionnaire, E = examination, NM = not mentioned, F = female, M = male

**Study**	**Country**	**Study design**	**Tool**	**Urban or rural**	**Setting**	**Sample size**	**Population**	**Age:Yrs**	**Gender**	**Response rate**
Mulimba 1990^19^	Nairobi	Retrospective	E	Urban	Private clinic	2201	Ortho patients	11–75	F/M	NM
Bezzaoucha 1992^21^	Algiers	Survey	Q	Rural	Community	6956	Residents	15 +	F/M	NM
Bwanahali et al 1992^22^	Zaire	Retrospective	Q	Rural	Hospital	169	OPD patients		F/M	NM
Harris 1993^20^	South Africa	Survey	Q	Urban	Clubs	110	Cricketers	15–35	M	90
Schierhout et al 1993^29^	South Africa	Cross-sectional	Q	Urban	Work	155	Factory workers	NM	F/M	100
Mijiyawa et al 2000^24^	Togo	Retrospective	Q	Rural	Hospital	9065	OPD patients	17–94	F/M	100
Omokhodion et al 2000^25^	Nigeria	Cross-sectional	Q	Rural	Hospital	74	Hospital staff	20–60	F/M	93
Worku 2000^11^	South Africa	Retrospective	Q	Rural	Community	4001	Mothers	NM	F	100
Wallner-Scholtfeldt et al 2000^30^	South Africa	Survey	Q	Urban	Work	196	Workers	23–59	M	64
Omokhodion 2002^26^	Nigeria	Cross-sectional	Q	Rural	Houses	900	Residents	20–85	F/M	100
Mbaye et al 2000^23^	Senegal	Cross-sectional	Q	Urban	Work	69	Workers		M	NM
Omokhodion et al 2003^15^	Nigeria	Cross-sectional	Q	Urban	Work	1285	Office workers	20–60	F/M	66
Igumbor et al 2003^31^	Zimbabwe	Cross-sectional	Q	Urban	Work	198	Physiotherapists	23–76	F/M	72
Omokhodion 2004^16^	Nigeria	Cross-sectional	Q	Urban	Community	474	Residents	20–82	F/M	100
Govender 2004^32^	South Africa	Survey	Q	Urban	Hospital	320	Nurses	20–62	F/M	68
Puckree et al 2004^33^	South Africa	Survey	Q	Urban	Schools	320	Scholars	11–14	F/M	55
Prista et al 2004^27^	Mozambique	Survey	Q	Rural/urban	Schools	204	Scholars	11–16	F/M	85
Fabunmi et al 2005^41^	Nigeria	Survey	Q	Rural	Farms	500	Farmers	25–84	F/M	100
Sanya et al 2005^42^	Nigeria	Cross-sectional	Q	Urban	Industry	604	Industrial workers	20–60	F/M	53
Bejia (Adol) et al 2005^34^	Tunisia	Cross-sectional	Q	Urban	Schools	622	Scholars	11–19	F/M	98
Jordaan et al 2005^28^	South Africa	Cross sectional	Q	Rural/Urban	Schools	1123	Scholars	13–18	F/M	89
Adedoyin et al 2005^35^	Nigeria	Survey	Q	Urban	Universities	1115	Computer users	NM	F/M	93
Bejia (hosp) et al 2005^36^	Tunisia	Survey	Q	Urban	Hospital	350	Hospital staff	18–60	F/M	100
Van Vuuren et al 2005^37^	South Africa	Cross-sectional	Q	Urban	Work	109	Workers	NM	M	96
Galukande et al 2005^38^	Uganda	Cross-sectional	Q	Urban	Hospital	204	Outpatients	19–86	F/M	100
Van Vuuren et al 2005^39^	South Africa	Cross-sectional	Q	Urban	Work	366	Workers	NM	NM	100
Van Vuuren et al 2006^40^	South Africa	Cross-sectional	Q	Urban	Work	366	Workers	NM	M	100

### Definition of low back pain

Ten studies (37%) provided a definition for LBP [21,26-28,30,32,33,36,38,41]. "Pain limited to the region between the lower margins of the 12^th ^rib and the gluteal folds" was a definition which appeared to be the most complete and thorough, leaving no opportunity for misinterpretation [38]. The definitions of LBP reported in the studies are listed in Table 4.

**Table 4:** LBP definitions of high-quality studies

**Author**	**Back pain definition**
Bezzaoucha 1992^21^	Existence of pain in the lumbar region
Wallner-Schlotfedlt et al 2000^30^	Pain in the lumbar region
Omokhodion 2002^26^	Graphic representation of lumbar area
Govender 2004^32^	Pain between the 12^th ^rib and gluteal fold
Puckree et al 2004^33^	Pain in specific region of the body
Prista et al 2004^27^	Pain in the lumbar area
Fabunmi et al 2005^41^	A condition of pain, aches, stiffness, or fatique localized to lower back or lumbosacral region of spine
Jordaan et al 2005^28^	Pain or discomfort in the lower part of your back
Bejia et al 2005^36^	Mechanical pain of the lower part of your back
Galukande et al 2005^38^	Pain limited to the region between the lower margins of the 12^th ^rib and the gluteal folds

### Treatment

Eight of the studies (33%) reported on the management of LBP [15,16,25,26,31,32,34,36] (Table 5). Medical doctors (general practitioners) and physiotherapists were the most common health professionals consulted by LBP sufferers in Africa, whilst analgesics and rest were the most common management strategies.

**Table 5:** LBP management

**Study(author)**	**Treatment type**	**Treatment reported**
Omokhodion et al 2000^25^	rest, analgesics	70% analgesics; 29% rest
Omokhodion 2002^26^	analgesics, orthodox health care personnel, non-orthodoxed personnel, patent medicine stored, traditional healers, drug peddlers, rest	Rest 80%; Analgesics 18% No treatment 42%
Omokhodion et al 2003^15^	health practitioner, rest	
Igumbor et al 2003^31^	rest, physician and other therapists	
Omokhodion 2004^16^	analgesics, health practitioner, anti-inflammatory drugs, clinic, hospital, chemist	Analgesics 61%; Seen medical practitioner 77%
Govender 2004^32^	medication, rest, physiotherapy, hospital admission, manipulation, acupuncture, surgery	Medication73%; Rest 59%; Physiotherapy 46% Hospital admission 26%
Bejia et al 2005^34^	Physiotherapy, medical officer	32% had treatment (Medical officer or physiotherapy);
Bejia et al 2005^36^	physiotherapy, self medication, surgery, rest, thermal water care	Medication 42%, Physiotherapy 15%, Surgery 0.0002%

### Methodological appraisal

The methodological quality scores of the included studies are reported in Table 6. As questionnaires were the main data collection instruments in the included studies, criterion 8 and 9 in the selected critical appraisal instrument were not applicable and omitted. However, an exception was made for the study done by Mulimba et al 1990, as it was the only study which used an examination [19]. For this instance, questions 7 and 8 were omitted, and in effect, question 9 was reinstated. Consequently, the total possible methodological quality score was 10. Considering that the mean methodological score was 71% (SD 20.06%), the authors determined arbitrarily that the threshold for acceptable study quality was 70%. Under this ruling, eighteen (67%) of the 27 studies [15,16,21,25-32,34,36-38,40-42] were deemed methodologically acceptable i.e. scoring at least 70% for methodological quality. The LBP prevalence data extracted from these methodologically sound studies were analyzed. Sensitivity analysis of the studies not found to be methodologically sound was done to determine if there would have been any difference in the results, had these studies been included.

**Table 6:** Critical appraisal of epidemiological studies

**Criterion no.**	**1**	**2**	**3**	**4**	**5**	**6**	**7**	**8**	**9**	**10**	**11**	**12**	**%**	**MA**
Mulimba 1990^19^	√	X	X	√	√	√	NA	NA	X	**X**	**X**	**X**	40	**N**
Bezzaoucha 1992^21^	√	X	√	√	√	√	X	NA	NA	√	X	**√**	70	**Y**
Bwanahali et al 1992^22^	X	X	√	X	X	√	x	NA	NA	x	x	x	20	**N**
Harris 1993^20^	X	X	√	√	√	√	X	NA	NA	X	√	**√**	60	**N**
Schierhout et al 1993^29^	√	√	√	√	√	√	X	NA	NA	X	X	**√**	70	**Y**
Mijiyawa et al 2000^24^	X	X	√	X	X	√	X	NA	NA	X	X	**√**	30	**N**
Omokhodion et al 2000^25^	√	X	√	√	√	√	X	NA	NA	X	√	**√**	70	**Y**
Worku 2000^11^	√	X	√	X	√	√	X	NA	NA	X	√	**√**	60	**N**
Wallner-Scholtfeldt et al 2000^30^	X	√	√	√	√	√	X	NA	NA	√	√	**√**	80	**Y**
Omokhodion 2002^26^	√	√	√	√	√	√	X	NA	NA	√	√	**√**	90	**Y**
Mbaye et al 2000^23^	X	X	√	√	√	√	X	NA	NA	X	√	**√**	60	**N**
Omokhodion et al 2003^15^	√	√	√	√	√	√	X	NA	NA	X	X	**√**	70	**Y**
Igumbor et al 2003^31^	√	√	√	√	√	√	X	NA	NA	X	√	**√**	80	**Y**
Omokhodion 2004^16^	√	√	√	√	√	√	X	NA	NA	X	√	**√**	90	**Y**
Go√ender 2004^32^	√	√	√	√	√	√	X	NA	NA	√	√	**√**	90	**Y**
Puckree et al 2004^33^	X	X	√	X	√	√	√	NA	NA	X	X	**√**	50	**N**
Prista et al 2004^27^	√	X	√	√	√	√	√	NA	NA	√	√	**√**	90	**Y**
Fabunmi et al 2005^41^	√	X	X	√	√	√	√	NA	NA	√	√	√	80	**Y**
Sanya et al 2005^42^	√	x	√	√	√	√	X	NA	NA	X	√	√	70	**Y**
Bejia (Adol) et al 2005^34^	√	√	√	√	√	√	√	NA	NA	√	√	**√**	100	**Y**
Jordaan et al 2005^28^	√	√	√	√	√	√	√	NA	NA	√	√	**√**	100	**Y**
Adedoyin et al 2005^35^	X	√	√	X	√	√	X	NA	NA	X	√	**√**	60	**N**
Bejia (hosp) et al 2005^36^	√	√	√	√	√	√	X	NA	NA	√	√	**√**	90	**Y**
Van Vuuren et al 2005^37^	√	√	√	√	√	√	√	NA	NA	X	√	**√**	90	**Y**
Galukande et al 2005^38^	X	√	√	√	√	√	X	NA	NA	√	√	**√**	80	**Y**
Van Vuuren et al 2005^39^	X	X	√	√	√	√	X	NA	NA	X	√	**√**	60	**N**
Van Vuuren et al 2006^40^	√	X	√	√	√	√	X	NA	NA	X	√	**√**	70	**Y**

√ = criterion fulfilled Y = Yes MA = methodologically accepted

X = criterion not fulfilled N = No NA = not applicable

### LBP prevalence

LBP prevalence was compared across only those studies which met the methodologically acceptable criteria (70% and higher scores). The definition of LBP was similar in these studies, this being generally "pain experienced in the lower back region". Two of these methodologically sound studies included male subjects only [30,37] and these results were reported separately. The rest of the studies included female and male subjects.

#### Point prevalence for high quality studies including males and females

**Figure 2:**
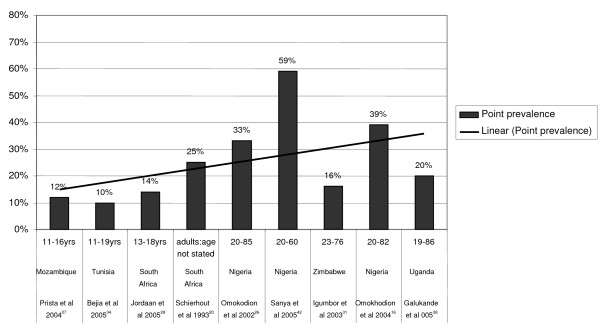
LBP point prevalence. LBP point prevalence ranged from 10% to 14% among adolescents, and 16% to 59%. The trend-line suggests that LBP point prevalence potentially increases with age.

Nine methodologically sound studies [26-29,31,34,38,42] provided point prevalence data. LBP point prevalence ranged from 10% to 14% among adolescents and 16% to 59% among adults. The mean LBP point prevalence among the adolescents was 12% and among the adults was 32%. The trend-line in Figure 2 suggests that LBP point prevalence potentially increases with age.

#### One-year prevalence for high quality studies including males and females

**Figure 3:**
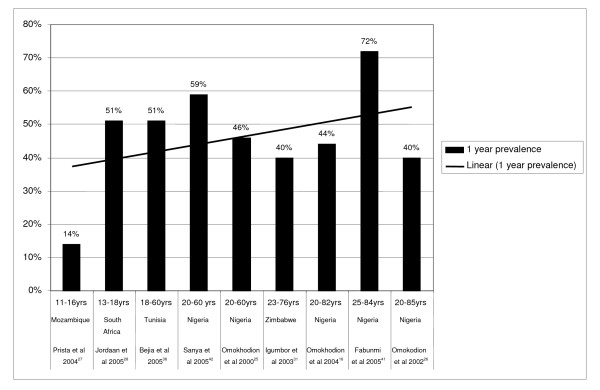
One-year LBP prevalence. The one year prevalence ranged from 14% to 72%. The trend-line suggests that one-year LBP prevalence potentially increases with age.

Nine studies [16,25-28,31,36,41,42] provided one-year LBP prevalence data. The one-year prevalence ranged from 14%-72%. The average one year prevalence among adolescents was 33% and among adults was 50% (Figure 3). The trend-line in Figure 3 suggests that one-year LBP prevalence potentially increases with age.

#### Lifetime prevalence for high quality studies including males and females

**Figure 4:**
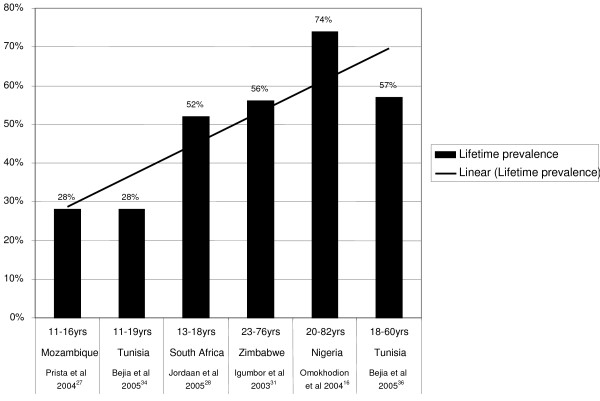
Lifetime LBP prevalence. The lifetime LBP prevalence ranged from 28% to 74%. The trend-line suggests that lifetime LBP prevalence potentially increases with age.

Six studies [16,27,28,31,34,36] provided lifetime LBP prevalence data, which ranged from 28% to 74%. The average lifetime prevalence among adolescents was 36% and among adults was 62% (Figure 4). The trend-line in Figure 4 suggests that lifetime LBP prevalence potentially increases with age.

#### Studies reporting on male LBP prevalence only

Considering the four studies which reported on only male subjects [20,23,30,37], the settings were a cricket club [20], a motor vehicle parts distribution centre [29], a manganese plant [37] and a public transport company [23]. Only two studies [20,30] reported the age ranges of subjects, collectively being 15–59 years. Only two of these studies were methodologically sound [30,37]. The prevalence of LBP in these two studies was 43% and 72% respectively [30,37].

### Sensitivity analysis

The methodological quality was less than 70% in 9 of the eligible studies [11,19,20,22-24,33,35,39]. The primary outcome of this review was LBP prevalence, and LBP point prevalence was provided by 7 of the 9 studies excluded due to methodological quality. The LBP point prevalence in these 7 studies ranged between 10–74% (M = 39%) among male and female adults (Table 7) [11,19,20,22,24,35,39]. The adult LBP point prevalence reported in the methodologically acceptable studies ranged between 16–59% (M = 32%). Limiting the analysis of studies based on methodological score therefore did not affect the LBP mean point prevalence significantly (all inclusive mean point LBP = 35.5%).

**Table 7:** LBP Prevalence from 9 studies with less than 70% methodological score

**Study**	**Point prevalence**	**One year prevalence**	**Lifetime prevalence**	**Population/setting**
Mulimba 1990^19^	10	-	-	Orthopaedic clinic
Bwanahali et al 1992^22^	47	-	-	Rheumatology clinic
Harris 1993^20^	62	-	-	In cricketers
Worku 2000^11^	10	-	-	Rural community/mothers
Mijiyawa et al 2000^24^	35	-	-	Rheumatology unit
Mbaye et al 2002^23^	-	-	-	Transport company
Puckree et al 2004^33^	-	-	-	School children
Adedoyin et al 2005^35^	74	-	-	Computer users
Van Vuuren et al 2005^39^	36	56	64	Steel industry

It was not possible to test for differences in annual and lifetime prevalence of LBP as only one of the excluded studies [39] reported on annual and lifetime LBP prevalence.

### Risk factors

**Figure 5:**
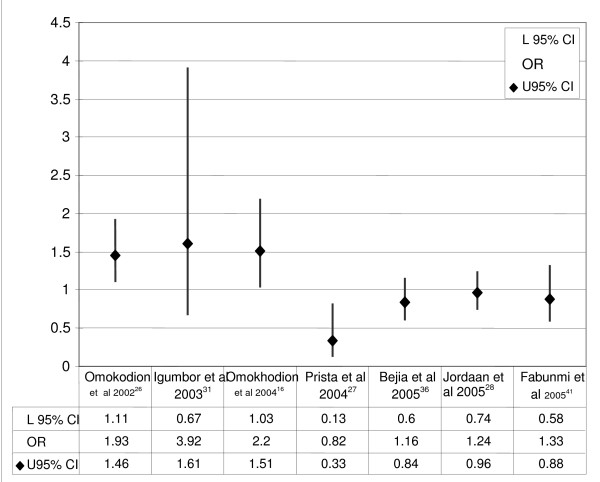
Odds ratios. Three of the seven studies cross one, indicating that the female gender is a significant risk factor for LBP.

Seven studies [16,26-28,31,34,41] provided sufficient data to determine associations with potential risk factors using odds ratios and 95% confidence intervals. Data pertaining to the total sample with LBP (males and females), as well as to males or females with LBP, was extracted, and odds ratios were calculated using EpiInfo StatCalc (version 3.3). Figure 5 reports on these odds ratios, indicating that the female gender was the only significant risk factor for LBP in three of the seven studies.

Five studies [16,27,28,34,36] of high methodological quality reported odds ratios for other LBP risk factors. The significant odds ratios were directly extracted from these publications and summarized in Table 8. Smoking [16,28] and a history of LBP [34,36] were each found to be risk factors for LBP.

**Table 8:** Odds ratio for LBP risk factors

**Author**	**Risk factor**	**Odds ratio(95% CI)**
Omokhodion et al 2004^16^	Past history of smoking	6.24 (1.33–29.23)
Jordaan et al 2005^28^	Smoking tobacco	1.69 (1.03–3.07)
Omokhodion et al 2004^16^	Farming	4.06 (1.24–12.95)
Prista et al 2004^27^	Urban school area	3.07 (0.99–9.48)
Prista et al 2004^27^	Walking >30 min	4.76 (1.61–14.28)
Bejia et al 2005^34^	School failure	2.6 (1.96–3.8)
Bejia et al 2005^34^	Football	3.07 92.15–5.1)
Bejia et al 2005^34^	Dissatisfaction with school chair	3.4 (2.24–5.29)
Bejia et al 2005 (adults)^36^	LBP history	18.6 (2.92–35.04)
Bejia et al 2005 (adults)^36^	LBP history	6.46 (1.86–17.52)
Omokhodion et al 2004^16^	History of trauma	4.14 (1.99–8.61)
Bejia et al 2005^34^	LBP family history	3.8 (2.94–5.92)
Bejia et al 2005 (adults)^36^	Psychological profile	1.93 (1.01–3.9)
Bejia et al 2005 (adults)^36^	Married/divorced	4.79 (1.56–22.57)

## Discussion

This is the first known systematic review to report on the findings of LBP prevalence and LBP risk factors among African populations. A global review published in 2000 suggested that only one African study was available for inclusion [8]. This current review reports a much larger group of relevant African studies (most published since 2000), of which 67% were methodologically sound. This review indicates that there is little difference in the prevalence of LBP among Africans compared with the prevalence of LBP in developed countries. The earliest publication in this review was written in 1990, which may indicate that LBP prevalence may be a relatively recent and emerging problem in Africa [19]. However this may also be an indication that publishable LBP research has only received resource support since the early 1990s, reflecting constraints on health research resources available on the African continent for orthopaedic research, as a result of urgent research into other health threats such as HIV/AIDS.

The most common population group studied was "workers". This finding is plausible given that there is a lack of legislation to support workers suffering from LBP to ensure that they receive optimal rehabilitation and support. In contrast, in western societies, legislation to promote spinal health protects workers from lumbar spine injury or pain is in place and is monitored by government bodies [43]. The lack of best practice rehabilitation methods for LBP which could prevent chronic pain and disability is evident in the reports of the most common treatment methods reported in the African research to treat LBP. Despite the increasing scientific evidence from meta-analyses that active rehabilitation involving exercise is most effective in reducing disability and LBP recurrence [44,45], the most common forms of management identified in this review were rest and analgesics. Well designed prevalence studies into African LBP, coupled with intervention studies to test the effectiveness of high quality interventions should be undertaken to inform and transform labor legislation policies to ensure better support for workers suffering from LBP.

'School scholars' were the second most common group studied by African researchers. This is viewed as a positive step, particularly with respect to setting the scene for primary prevention of LBP. The results of this review indicate that there is reason for concern regarding LBP prevalence in adolescents. Of further concern is that a "history of LBP" as reported in many western societies as a causal agent of LBP, may also be an important predictor of LBP among Africans. This implies that much LBP experienced by young people may manifest into chronic LBP in adulthood. Chronic LBP is costly to manage due to recurrent and debilitating nature of the condition. The findings of this review indicate that primary prevention should be considered an African priority due to the already constrained economic resources for overall health care [3,5,8]. Another factor that may be increasing the prevalence of LBP among young African people is the widespread introduction of information technology systems in African schools. For instance, in the Western Cape of South Africa, all or most schools will probably be equipped with computer laboratories by the year 2012 for curriculum delivery in an attempt to compensate for the increasing shortage of school educators. While the use of technology may be best practice for educating young people, it also introduces the likelihood of young people developing poor postural habits unless they are specifically instructed otherwise. The school setting may therefore be appropriate to teach young people good spinal health habits, and future research should incorporate spinal health promotion strategies for schools in Africa.

The mean LBP point prevalence among the African adolescents was 12% and among the African adults 32% (range 10% to 59%). This finding negates any assumptions that LBP point prevalence is lower in the developing world than developed societies, as the range of LBP point prevalence among western societies is also reported to range between 12% and 33% [8,37]. This revelation supports the findings of the global burden of disease studies which predict that the greatest increases in LBP prevalence will be in developing nations [6]. Unfortunately, the data obtained from the studies included in this review is insufficient to ascertain the trend of LBP over more than 2 decades, as the earliest methodologically acceptable study reporting point prevalence was published in 1993 [29]. However, with over 80% of the included studies published after the year 2000, there will be the capacity in a few years time to predict African LBP prevalence trends with some certainty.

The one-year LBP prevalence among Africans ranged from 14% to 72%. The one-year prevalence among Western societies is reported to be between 20% and 62% [8]. Therefore it appears that the one-year prevalence estimated among Africans correlates with the one-year LBP prevalence in Western societies. Similarly, comparable findings were observed for lifetime prevalence estimates as African lifetime prevalence ranged from 28% to 74%, whilst lifetime prevalence in Western societies ranged from 30% to 80% [37]. Advances in technology and the mechanization of industries in African countries may therefore be reflected in the high one-year and lifetime prevalence of LBP among Africans, reported in the past decade of research. However, there is insufficient data on rural populations as only three [25,26,41] of the 18 methodological acceptable studies provided data exclusively on rural populations. The prevalence reported in these studies is comparable to reported urban population prevalence, and may reflect that the considerable physical activities required for rural (farming) activities may be a risk factor for LBP [25,26,41]. The study by Omokhodion (2004) illustrates that farming activities increase the odds of suffering LBP by four, compared with individuals not exposed to farming activities [16]. These findings related to one-year and lifetime prevalence, and further illustrates that LBP among all Africans is of concern. Further research into the most effective strategies to manage and prevent LBP is warranted.

Nine of the studies were excluded from data analysis due to the fact that they did not score above 70% methodologically. The most common shortcomings were a clear definition of LBP, lack of adequate representation of the population, lack of detail on the instrument used, and lack of using a reliable and valid questionnaire to collect data (Tables 1 and 6). These methodological shortcomings have ramifications into the validity of the study findings. For instance if a questionnaire is not culturally appropriate for a specific African population, it could mean that inappropriate or incomplete questions were asked pertaining to potential risk factors, or that respondents did not answer some questions as they were considered culturally insensitive or inappropriate. The mean response rate to the African studies included in this review was 88%, which correlates with the mean response rate of 81% reported in the systematic review by Walker [8]. The reason for poor response rates in six [15,30-33,42] of the African studies was often cited as incomplete questionnaires, and this may be because the questionnaire content and language was not culturally acceptable. One critical area to be addressed by African researchers is to ensure that data collection tools are valid for specific target populations, and are reliably answered.

## Conclusion

The findings of this review indicate that the prevalence of LBP among Africans may be comparable to that reported in research undertaken in developed nations. Therefore further research into the identification, prevention and best practice management of LBP is also necessary in African countries. Furthermore, there is a clear mandate for African researchers to improve the methodological quality of their LBP epidemiological studies, considering the reliability and validity of measurement instruments, and agreeing on a standard definition of the condition. Possible limitations to this study could be that certain African journals were inaccessible on electronic databases, as they were published locally, were only available in the specific African country and not obtainable in South Africa. There was difficulty contacting the researchers and libraries in these countries.

## Competing interests

The author(s) declare that they have no competing interests.

## Authors' contributions

Quinette Louw conceived the study idea, designed the review methodology, conducted the critical appraisal of the studies and drafted the manuscript.

Linzette Morris developed the search strategies, conducted the searches, conducted the critical appraisal of the studies and prepared the final manuscript for publication.

Karen Grimmer-Somers assisted in designing the review methodology.

All authors read and approved the final manuscript.

## Appendix A: Database search strategies

PUBMED

1. "back pain" [MeSH Major topic]

2. "Africa" [MeSH]

3. #1 AND #2

4. "physical therapy(Speciality)" [MeSH]

5. #3 AND #4

6. "prevalence" [MeSH Major topic]

7. #3 AND #6

8. "Epidemiology" [MeSH Major topic]

9. #3 AND #8

10. "rehabilitation" [MeSH Major topic]

11. #5 AND #8

12. "Primary Prevention" [MeSH Major Topic]

13. #3 AND #12

ProQuest

1. Africa

2. back pain

3. spinal pain

4. #1 AND #2

AfricaWide

1. back pain

2. (#1 AND physiotherapy)

3. (#1 AND prevention)

4. (#1 AND rehabilitation)

5. (#1 AND prevalence)

6. (#1 AND conditions)

7. back conditions

8. (spine AND pain)

9. spine conditions

SA ePublications

1. kw: back pain

2. kw: spinal pain

3. kw: spine

4. kw: physiotherapy

5. (kw: back pain) and kw: physiotherapy

6. kw: back rehabilitation

7. kw: back conditions

8. ((((((kw: (spine)) or (kw: (back conditions))) or (kw: (back rehabilitation))) or (kw: (physiotherapy))) or (kw: (spinal pain))) or (kw: back pain))

Psycinfo

1. ("Back-Pain" in MJ, MN) or ("Back-Anatomy" in MJ, MN)

2. (africa)

3. spinal pain

4. "Epidemiology-" in MJ, MN

5. "Rehabilitation-" in MJ, MN

6. #1 and #2

7. #1 and ("Epidemiology-" in MJ, MN)

8. #5 and #2

9. ("Rehabilitation-" in MJ, MN) and #1

CiNAHL and ScienceDirect

1. (MM "Back pain+") or (MM "Low Back Pain") or (MM "Back Injuries+) or (MM "Back")

2. Africa

3. #1 AND #2

4. (MH "Spinal Injuries")

5. #2 AND #4

6. (MM "Preventative Health Care")

7. (MH "Rehabilitation")

8. #4 AND #7

SportsDiscus

1. (DE "BACK PAIN" OR DE "LOW BACK PAIN)

2. (DE "AFRICA")

3. #1 AND #2

4. (DE "PHYSICAL THERAPY" OR DE "BALNEOLOGY" OR DE "BATH" OR DE "CHIROPRACTIC" OR DE "CONTINUOUS PASSIVE MOTION" OR DE "DIATHERMY" OR DE "ELECTROTHERAPY" OR DE "HYDROTHERAPY" OR DE "INVERSION THERAPY" OR DE "MAGNETIC FIELD THERAPY" OR DE "MASSAGE" OR DE MYOFASCIAL RELEASE" OR DE "ORTHOPEDIC MANIPUALTION" OR DE "PROPRIOCEPTIVE NEUROMUSCULAR FACILITATION" OR DE "ROLFING" OR DE "SAUNA" OR DE "THERMOTHERAPY" OR DE "WATER RUNNING")

5. (DE "PREVENTION")

6. (DE "TREATMENT")

7. (DE "REHABILITATION")

8. (DE "EPIDEMIOLOGY")

9. (DE "ETIOLOGY")

10. #3 AND #4

## Pre-publication history

The pre-publication history for this paper can be accessed here:


